# Residual Disease Burden Among European Patients With Inflammatory Bowel Disease: A Real-World Survey

**DOI:** 10.1093/ibd/izae119

**Published:** 2024-06-07

**Authors:** Johan Burisch, Ailsa Hart, Andreas Sturm, Christine Rudolph, Rachael Meadows, Anna Jus, Fatima Dawod, Haridarshan Patel, Alessandro Armuzzi

**Affiliations:** Gastrounit, Medical Division, Copenhagen University Hospital – Amager and Hvidovre, Hvidovre, Denmark; Copenhagen Center for Inflammatory Bowel Disease in Children, Adolescents and Adults, Copenhagen University Hospital – Amager and Hvidovre, Hvidovre, Denmark; Department of Clinical Medicine, Faculty of Health and Medical Sciences, University of Copenhagen, Copenhagen, Denmark; Inflammatory Bowel Disease Unit, St Mark’s Hospital, Harrow, United Kingdom; Department of Internal Medicine - Gastroenterology, German Red Cross Hospital Berlin, Berlin, Germany; Galapagos NV, Leiden, Netherlands; Employee of Alfasigma S.p.A at the time of publication; Adelphi Real World, Bollington, United Kingdom; Galapagos NV, Leiden, Netherlands; Employee of Alfasigma S.p.A at the time of publication; Adelphi Real World, Bollington, United Kingdom; Galapagos NV, Mechelen, Belgium; Inflammatory Bowel Diseases Center, IRCCS Humanitas Research Hospital, Rozzano, Italy; Department of Biomedical Sciences, Humanitas University, Pieve Emanuele, Italy

**Keywords:** inflammatory bowel disease, health-related quality of life, real-world survey, patient-reported outcomes, comprehensive disease control

## Abstract

**Background:**

Understanding disease burden is imperative for improving inflammatory bowel disease (IBD) management. This real-world survey investigated residual disease burden and treatment satisfaction among European patients with moderate-to-severe ulcerative colitis (UC) and Crohn’s disease (CD).

**Methods:**

The Adelphi Real World IBD Disease Specific Programme was a multinational, cross-sectional survey with retrospective collection of patient- and physician-reported data on disease burden and management. Between October 2020 and March 2021, participating gastroenterologists recruited their next 7 (UC) and 8 (CD) eligible patients and reported demographics and clinical characteristics. Patients completed symptom, health-related quality of life (HRQoL), and treatment satisfaction questionnaires. Data were adjusted for confounding variables and compared between patients in remission (clinical remission, endoscopic remission, or both) and not in remission.

**Results:**

Overall, 1040 patients (UC, n = 502; CD, n = 538) were included. Although most patients were in remission (UC, 66.1%; CD, 69.5%), most still reported symptoms (UC, 63.7%; CD, 74.1%), including flatulence, fatigue/tiredness, and abdominal pain/distension. In UC, there were no significant differences in the likelihood of experiencing 7 of 23 symptoms between patients in remission and not in remission. In CD, there was no significant difference in 19 of 23 symptoms between patients in remission and not in remission. Several symptoms were significantly associated with reduced HRQoL. HRQoL was significantly better among patients in remission than not in remission.

**Conclusions:**

Patients with IBD, both in remission and not in remission, experience residual symptoms that impair HRQoL. Comprehensive endpoints, incorporating HRQoL and patients’ perspectives, and improved treatments are needed to address residual disease and patients’ needs.

**Key Messages**

**What is already known?**

Although multiple advanced therapies exist for inflammatory bowel disease (IBD), it remains incurable and impacts health-related quality of life (HRQoL).

**What is new here?**

This extensive, real-world survey of over 1000 European patients with IBD highlights substantial treatment dissatisfaction and residual disease burden, including symptoms and impaired HRQoL, among patients in remission.

**How can this study help patient care?**

This study identifies areas of unmet need and provides rationale for the development of novel therapeutic approaches and new efficacy endpoints that include patients’ HRQoL. This could enable more comprehensive disease control in patients with IBD.

## Introduction

Inflammatory bowel disease (IBD) is a chronic, idiopathic, immune-mediated disease. The 2 main forms of IBD are ulcerative colitis (UC) and Crohn’s disease (CD). Patients with IBD experience chronic inflammation of the gastrointestinal tract, resulting in abdominal pain, recurring (and sometimes bloody) diarrhea, weight loss, and fatigue.^1^ Patients can experience progressive bowel damage and increased risk of malignancies and/or the need for intestinal resections.^2^ In addition to gastrointestinal symptoms, the widespread impact of IBD on psychological, physical, sexual, and social well-being dramatically reduces health-related quality of life (HRQoL).^3-5^

As IBD is currently incurable, the aim of treatment is to induce and maintain disease remission. Treatment targets include amelioration of symptoms, endoscopic healing, and improvements in HRQoL.^6–9^ Current approaches include corticosteroids, conventional treatments such as aminosalicylates and thiopurines, and advanced therapies such as inhibitors of tumor necrosis factors, integrins, interleukins, and Janus kinases.^10^ However, a considerable number of patients demonstrate a lack or loss of response to current therapies.^11^ Even among those treated with advanced treatments, around 50% of patients do not experience sufficient response,^12,13^ and current treatments are associated with adverse events that limit patient adherence.^11,14^ Therefore, despite multiple treatment options, there remains a significant disease burden in patients with IBD, encompassing wide-ranging symptoms, impaired HRQoL, and an elevated risk of requiring surgery.^15,16^

Recognizing the complex residual disease burden experienced by patients with IBD is imperative for developing informed, targeted, and effective treatment strategies that could improve patient care.^17^ This includes understanding symptom burden in patients both in remission and not in remission, understanding the factors that drive treatment seeking, acknowledging current therapeutic gaps, and evaluating patient expectations and requirements. Real-world surveys of patients with IBD have shown that active disease is associated with impaired HRQoL and reduced productivity compared with remission or with less active disease.^18,19^ Indeed, these patient-reported outcomes (PROs) have been identified by patients as key targets for effective treatments.^9^ Furthermore, evaluating the association between different symptoms and HRQoL has allowed real-world surveys to steer research efforts by identifying potential new treatment targets.^20^ Real-world surveys are also effective tools for monitoring the impact of new therapeutics by measuring residual disease burden outside of a clinical trial setting.^21^

This study investigated the disease burden among patients in Europe with moderate-to-severe IBD, including their residual symptoms, HRQoL, and treatment satisfaction.

## Methods

### Study Design

The Adelphi Real World IBD Disease Specific Programme (DSP) was a large, multinational, cross-sectional survey conducted in routine clinical practice. The survey described current disease management, disease burden impact, and associated treatment effects (clinical and physician-perceived) in IBD.^22^ The present study used data collected in France, Germany, Italy, and Spain between October 2020 and March 2021.

All participating gastroenterologists recruited patients seen in clinical practice. Gastroenterologists collected data on baseline demographics, disease history (including retrospective data on patients’ symptoms prior to the initiation of their current treatment), and clinical characteristics. Patients had the option of completing a 20-minute questionnaire about their personal experiences with IBD. A complete description of the validated methods of the DSP methodology has been previously published.^22-24^

### Ethical Considerations

Data collection was undertaken in line with European Pharmaceutical Market Research Association guidelines and as such this study did not require ethics committee approval.^25^ Each survey was performed in full accordance with the relevant legislation at the time of data collection, including the Health Information Technology for Economic and Clinical Health Act legislation.^26^ Ethical exemption was obtained from the Western Institutional Review Board, study protocol number 1-1238963-1. All patients provided informed consent to take part in the survey. Data were collected in such a way that ensured that patients and physicians could not be identified, and all data were pseudo-anonymized both at the point of collection and upon receipt by the researchers. Data were aggregated before being shared with the subscriber and/or being prepared for publication.

### Inclusion Criteria

Physicians were identified by local fieldwork teams from publicly available lists and contacted for further eligibility assessment. Eligible physicians were those in secondary gastroenterology services (hospitals, clinics, or offices) and who were willing to adhere to the study protocol. Physician location was also considered to ensure a wide geographic spread.

Each gastroenterologist had agreed to participate in the full IBD DSP and collected data on the next up to 7 (UC) and up to 8 (CD) consecutive eligible patients who they saw in consultations. Patients included were 18 years of age or older, had a physician-confirmed diagnosis of UC or CD, and were not participating in any clinical trials. There were no additional criteria for patients with CD. Physicians were asked to select the patient’s disease severity status at initiation of their current treatment from 3 categories (mild, moderate, or severe) based on their clinical opinion. For this study analysis, patients were drawn from the DSP if they had UC or CD that was classed as moderate or severe at initiation of current treatment, and if they had completed a patient self-completion form (see Data Collection and Outcome Measures).

### Data Collection and Outcome Measures

Once a patient had been recruited, gastroenterologists completed a 25-minute patient record form, providing data on demographics and clinical characteristics, and a full medical history. This questionnaire was completed online and anonymized at the point of data entry. Data on treatment satisfaction were also collected by asking patients to select how satisfied they were with their current treatment (“How satisfied are you with how well your medication controls your condition?”) from 4 options (“satisfied and believe this is the best control that can be achieved”; “satisfied, but believe better control can be achieved”; “not satisfied, but believe this is the best control that can be achieved”; or “not satisfied, and believe better control can be achieved”).

Patients completed their questionnaires either online or using pen and paper, at their preference. Patients completed their questionnaires independently from physicians and returned them anonymously. For symptom data, patients were provided with a list of symptoms and asked to indicate the ones that they experienced. Of those, patients were asked to select the 3 most bothersome symptoms. Patients were also asked to select which symptoms they were experiencing prior to initiation of their current treatment.

As part of the survey, patients completed several patient-reported outcome (PRO) measures including the EQ-5D questionnaire,^27^ the Short Inflammatory Bowel Disease Questionnaire (SIBDQ),^28^ and the Work Productivity and Activity Impairment (WPAI) questionnaire.^29^ The EQ-5D is a HRQoL questionnaire that comprises a visual analog scale (VAS) and a descriptive section. The VAS allows patients to report overall current health state, from the best imaginable health state to the worst imaginable health state. The EQ-5D descriptive section defines health in 5 dimensions: walking around (mobility), washing and dressing oneself (self-care), usual activities, pain/discomfort, and anxiety/depression. Patients report how problematic they find each domain, with 5 levels of severity: no problem, slight problem, moderate problem, severe problem, or unable to function normally. The SIBDQ is also used to assess HRQoL and is scored from 10 to 70, indicating poor to good HRQoL. It consists of a total score, and scores for 4 individual domains: systemic symptoms, social symptoms, bowel symptoms, and emotional symptoms. A licensing agreement from McMaster University was obtained for use of the SIBDQ.^28^ The impact of disease on daily functioning was measured by the WPAI questionnaire, which includes 4 components, each expressed as percentage impairment: absenteeism, presenteeism, work productivity loss, and activity impairment.

Patients were stratified by their type of IBD (UC or CD). Within each cohort, patients’ disease burden was compared between those in remission and those not in remission. Remission status was reported by the gastroenterologist at the time of consultation. Patients with UC were considered to be in remission if their Mayo stool frequency subscore was 0 or 1, rectal bleeding subscore was 0, and endoscopic subscore was 0 or 1. Patients who had any of the following were considered to not be in remission, even if their 2 other subscores were normal: a stool frequency subscore of 2 or more, a rectal bleeding subscore of 1 or more, or an endoscopic subscore of 2 or more. In patients with CD, patients were categorized into 5 categories of remission: (1) clinical/symptomatic remission, but with full mucosal healing not achieved; (2) clinical/symptomatic remission, but mucosal healing currently unknown; (3) full mucosal healing, but symptoms remained; (4) clinical/symptomatic remission and full mucosal healing; and (5) symptoms remained and mucosal healing not achieved. When variables were compared between those in remission and those not in remission, patients in categories 1 to 4 (clinical/symptomatic remission, endoscopic remission, or both) were considered to be in remission. Patients in category 5 (not in clinical/symptomatic remission or endoscopic remission) were considered as not in remission.

In addition to remission status, disease burden was compared between those who were satisfied with their treatment (“satisfied and believe this is the best control that can be achieved”) and those who were not (“satisfied, but believe better control can be achieved”; “not satisfied, but believe this is the best control that can be achieved”; or “not satisfied, and believe better control can be achieved”).

### Statistical Analysis

Continuous variables were assessed by descriptive statistics and included mean, standard deviation, median, quartiles, and 95% confidence intervals. Categorical variables were reported as the number of patients being described by a particular variable and as a percentage of total patients. No imputation was used for missing data.

The probability of symptom occurrence at the time of consultation (based on the mean response in each group) and self-reported HRQoL (EQ-5D [Germany tariff] and SIBDQ) were compared between patients in remission and those not in remission. Propensity score matching (PSM) was used to adjust for confounding variables. Scores were estimated using a logistic regression model adjusting for covariates including age, sex, body mass index (BMI), time since diagnosis, time since start of current treatment, and disease severity at that time. Each patient in the remission group was matched to a patient in the nonremission group. The balance of the match was assessed by calculating standardized mean differences; a standardized mean difference of between −10% and +10% (inclusive) was considered indicative of adequate balance. PSM generated an adjusted mean response in each subgroup. The mean response for symptom data ranged between 0 (did not experience a symptom) and 1 (did experience a symptom). The mean response for HRQoL data was the adjusted mean score of each instrument. Adjusted mean symptom and HRQoL responses were compared between patients in remission and those not in remission using standard bivariate statistics (*t* test and analysis of variance [ANOVA]). Symptom occurrence prior to initiation of current treatment was also compared between patients in remission and those not in remission at the time of the consultation, without PSM adjustment. For all analyses, a *P* value < .05 was deemed statistically significant. There were no formal hypotheses, so no formal power calculation or determination of sample size was required.

Among patients with CD who were reported to be in clinical/symptomatic remission and who were reported to have mucosal healing, some had mucosal healing confirmed by endoscopy, whereas some did not. To assess the validity of the physician’s report of mucosal healing without endoscopy, an additional sensitivity analysis was performed to evaluate the impact of including endoscopic assessment in the definition of remission. Among those who were in endoscopic remission, demographics, clinical profiles, PROs, and occurrence of symptoms at the time of consultation were compared between those who had undergone endoscopy to confirm mucosal healing and those who had not undergone endoscopy.

In addition, individual linear regressions were conducted on EQ-5D, SIBDQ, and WPAI data, and included the 15 most reported symptoms as independent variables, with the aim of determining any multivariable associations between PROs and symptoms. Descriptive statistics were also used to compare the probability of reporting different symptoms between patients who were satisfied or not satisfied with their treatment. All statistical analysis was carried out in Stata Statistical Software release 15 (StataCorp LP, College Station, TX).

## RESULTS

### Patient Demographics and Clinical Characteristics

In total, 131 gastroenterologists reported on 502 patients with moderately to severely active UC, and 133 gastroenterologists reported on 538 patients with moderate-to-severe CD; patient demographics are shown in Supplementary Table 1. Patients were located across France (UC, n = 96; CD, n = 85), Germany (UC, n = 219; CD, n = 231), Italy (UC, n = 78; CD, n = 81), and Spain (UC, n = 109; CD, n = 141). Patients had a mean age of 38.9 (UC) and 38.3 (CD) years, were 46.0% (UC) and 53.5% (CD) male, and had a mean BMI of 23.3 (UC) and 23.5 (CD) kg/m^2^. Most patients (UC 76.7%; CD 70.9%) had received 2 or more lines of treatment (Supplementary Table 1). Overall, 60.6% of patients with UC (56.3% of those in remission and 68.8% of those not in remission) and 57.1% of patients with CD (58.0% of those in remission and 54.9% of those not in remission) were receiving advanced therapy at the time of consultation. Among patients with UC, 11.2% of patients (8.1% of those in remission and 17.1% of those not in remission) had anxiety and 5.2% of patients (3.3% of those in remission and 8.8% of those not in remission) had depression, as reported by the physician. Among patients with CD, 12.3% of patients (8.5% of those in remission and 20.7% of those not in remission) had anxiety and 7.8% of patients (4.0% of those in remission and 16.5% of those not in remission) had depression.

The results of the PSM on baseline covariates are found in Supplementary Table 2. The sensitivity analysis conducted in patients with CD in remission revealed no differences in patient demographics and clinical characteristics between those who had undergone endoscopy to confirm mucosal healing and those who had not undergone endoscopy (Supplementary Table 3).

### Symptoms

**Figure 1.**
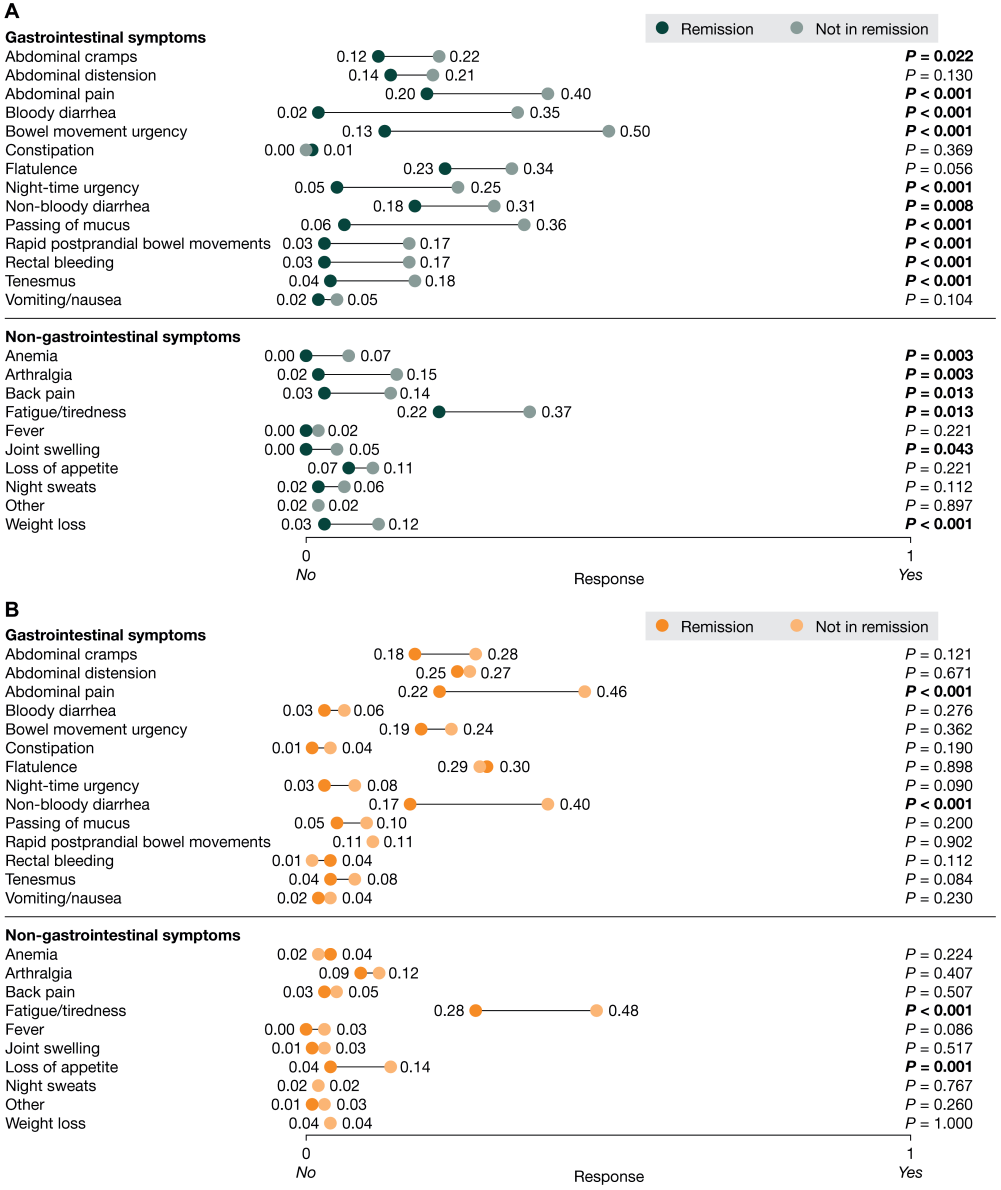
Adjusted mean response to whether or not a symptom was experienced at the time of consultation (1 = yes, 0 = no) among patients in remission and not in remission in (**A)** patients with ulcerative colitis and **(B)** patients with Crohn’s disease. Data are shown for patients with complete data for all outcomes and potential confounding variables (for ulcerative colitis, n = 271 and n = 147 for those in remission and not in remission, respectively; for Crohn’s disease, n = 299 and n = 128 for those in remission and not in remission, respectively). The *P* values demonstrate the significance of differences in adjusted mean response between patients in remission and those not in remission; *P* values in bold indicate statistical significance (< .05). Data were adjusted for age, sex, body mass index, time since inflammatory bowel disease diagnosis, time since the start of current treatment initiation, and disease severity at the time of current treatment initiation, using propensity score matching.

The adjusted mean response to whether a symptom was experienced or not at the time of consultation in patients in remission vs those not in remission are shown in Figure 1 (1 = yes, 0 = no). Among patients in remission, this ranged from 0.00 to 0.23 (UC) and from 0.00 to 0.30 (CD). Among patients not in remission, it ranged from 0.00 to 0.50 (UC) and from 0.01 to 0.48 (CD). In UC, patients not in remission were significantly more likely to experience 16 of the 23 symptoms assessed than patients in remission (all *P < *.05). In UC, the 3 symptoms with the largest difference in response between the remission and non-remission groups were bowel movement urgency, bloody diarrhea, and passing of mucus (all *P < *.001). The likelihood of experiencing the other 7 symptoms, including gastrointestinal symptoms such as abdominal distension and flatulence and non-gastrointestinal symptoms such as loss of appetite, was similar between those in remission and those not in remission (all *P* > .05). In CD, 4 out of the 23 symptoms assessed (abdominal pain, fatigue/tiredness, loss of appetite, and non-bloody diarrhea) were significantly more likely in patients not in remission than those in remission (*P* < .05). The remaining symptoms, including gastrointestinal symptoms such as abdominal cramps, abdominal distension, and bloody diarrhea, and non-gastrointestinal symptoms such as anemia, arthralgia, and weight loss, were similarly likely to occur among those in remission and not in remission (all *P* > .05). All symptoms, other than anemia (UC), fever (UC and CD), and joint swelling (UC), had an adjusted likelihood of > 0 among patients in remission, indicating that they were experienced to some degree despite remission.

The proportions of patients who reported each symptom prior to initiation of current treatment are shown in Supplementary Figure 1. Among patients in remission at the time of consultation, the proportion who reported experiencing each symptom prior to initiation of their current treatment ranged from 0% to 68% (UC) and from 0.5% to 79.7% (CD). For those not in remission, the proportion who reported experiencing each symptom ranged from 0% to 72.8% (UC) and from 0.6% to 81.9% (CD). Among the symptoms experienced prior to the initiation of current treatment in patients with UC, 9 out of the 26 symptoms assessed were reported by a significantly higher proportion of those not in remission at the time of consultation compared with those who were in remission (bowel movement urgency, flatulence, night-time urgency, passing of mucus, rapid postprandial bowel movements, rectal bleeding, vomiting/nausea, fatigue/tiredness, and weight loss). Among the symptoms experienced prior to the initiation of current treatment in patients with CD, 2 of the 27 symptoms assessed were reported by a significantly higher proportion of those not in remission at the time of consultation compared with those who were in remission (fatigue/tiredness and night sweats). In addition, bloody diarrhea was reported by a significantly higher proportion of patients with CD who were in remission at the time of consultation compared with those not in remission at the time of consultation.

Among patients in remission, 63.7% (UC) and 74.1% (CD) reported symptoms at the time of consultation. Of patients not in remission, 95.8% (UC) and 96.2% (CD) reported symptoms (Supplementary Table 1). Fatigue/tiredness (UC and CD), abdominal pain (UC and CD), bowel movement urgency (UC), and flatulence (CD) were the 3 symptoms experienced by the numerically highest proportions of patients (Supplementary Table 4). Fatigue/tiredness was reported by 26.9% of patients with UC (20.5% of those in remission and 39.4% of those not in remission) and 31.4% of patients with CD (26.5% of those in remission and 42.5% of those not in remission). Furthermore, 25.1% of patients with UC and 32.0% of patients with CD identified fatigue/tiredness as one of the most bothersome symptoms. Abdominal pain was reported by 25.7% of patients with UC (18.3% of those in remission and 40.0% of those not in remission) and 31.4% of patients with CD (23.1% of those in remission and 50.0% of those not in remission). It was identified as one of the most bothersome symptoms by 23.1% of patients with UC and 31.8% of patients with CD. Among patients with UC, bowel movement urgency was experienced by 25.5% of patients (13.7% of those in remission and 48.5% of those not in remission), and 30.5% of patients identified it as one of the most bothersome symptoms. Among patients with CD, flatulence was experienced by 27.2% of patients (27.0% of those in remission and 27.5% of those not in remission), and 24.2% of patients identified it as one of the most bothersome symptoms. The full list of symptoms and their bothersomeness can be found in Supplementary Table 4.

The proportion of patients with CD in remission who reported different residual symptoms at the time of consultation, separated by whether or not they underwent endoscopy to confirm mucosal healing, are shown in Supplementary Table 5. Of patients in remission, a lower proportion of those who underwent endoscopy to confirm mucosal healing experienced abdominal pain and fatigue than those who had not undergone endoscopy to confirm mucosal healing (ANOVA and/or *t* test, *P = *.013 and *P = *.041, respectively). Nevertheless, for most symptoms, the differences among those in remission between those who had undergone endoscopy to confirm mucosal healing and those who had not undergone endoscopy were not statistically significant.

### Health-Related Quality of Life

HRQoL was generally similar between patients with UC and patients with CD (Table 1). Mean SIBDQ scores were 52.3 (UC) and 51.3 (CD), and mean EQ-VAS scores were 76.5 (UC) and 75.1 (CD). Numerically similar proportions of patients with UC and CD reported more than slight problems with each of the EQ-5D domains. The levels of WPAI-measured work and activity impairment were also similar among patients with UC and patients with CD.

**Table 1.** Patient-reported outcomes.

Variable	Patients with UC (n = 502)	Patients with CD (n = 538)
**EQ-5D**		
** EQ-VAS**	**n = 502**	**n = 536**
Mean ± SD	76.5 ± 16.9	75.1 ± 16.1
** Mobility domain**	**n = 502**	**n = 533**
Slight problems	58 (11.6)	49 (9.2)
More than slight problems^a^	18 (3.6)	17 (3.2)
** Self-care domain**	**n = 497**	**n = 532**
Slight problems	34 (6.8)	31 (5.8)
More than slight problems^a^	6 (1.2)	5 (0.9)
** Usual activities domain**	**n = 500**	**n = 533**
Slight problems	90 (18.0)	123 (23.1)
More than slight problems^a^	54 (10.8)	42 (7.9)
** Pain/discomfort domain**	**n = 499**	**n = 537**
Slight pain/discomfort	190 (38.1)	240 (44.7)
More than slight pain/discomfort^a^	89 (17.8)	104 (19.4)
** Anxiety/depression domain**	**n = 500**	**n = 535**
Slight anxiety/depression	151 (30.2)	201 (37.6)
More than slight anxiety/depression^a^	88 (17.6)	86 (16.1)
**SIBDQ total score**	**n = 493**	**n = 526**
Mean ± SD	52.3 ± 12.8	51.3 ± 11.7
**WPAI**		
** Overall work impairment**	**n = 242**	**n = 280**
Mean ± SD, %	20.4 ± 22.3	22.3 ± 22.5
** WPAI absenteeism**	**n = 262**	**n = 287**
Mean ± SD, %	9.8 ± 25.6	5.2 ± 15.8
** WPAI presenteeism**	**n = 288**	**n = 317**
Mean ± SD, %	17.9 ± 19.6	20.0 ± 19.5
** WPAI activity impairment**	**n = 486**	**n = 519**
Mean ± SD, %	24.6 ± 24.8	25.0 ± 22.9

Values are n (%), unless otherwise indicated. For a given variable, cohort size is indicated if data were not available for all patients.

Abbreviations: CD, Crohn’s disease; SIBDQ, Short Inflammatory Bowel Disease Questionnaire; UC, ulcerative colitis; WPAI, Work Productivity and Activity Impairment.

^a^Includes moderate problems with, severe problems with, and completely unable to perform normal functions.

Most patients were in full- or part-time employment (UC, n = 336 of 491 [68.4%]; CD, n = 352 of 533 [66.0%]). In the past 3 months, patients had taken a mean of 8.5 days (6.3 days for those in remission and 11.3 days for those not in remission) and 7.7 days (8.0 days for those in remission and 7.3 days for those not in remission) off work owing to their UC and CD, respectively. Furthermore, 11.0% of those with UC (8.1% of those in remission and 16.5% of those not in remission) and 10.8% of those with CD (9.9% of those in remission and 12.8% of those not in remission) had reduced their working hours owing to their disease. In addition, 9.8% of those with UC (5.7% of those in remission and 17.6% of those not in remission) and 11.2% of those with CD (8.6% of those in remission and 17.1% of those not in remission) had stopped working altogether (Supplementary Table 1).

**Figure 2.**
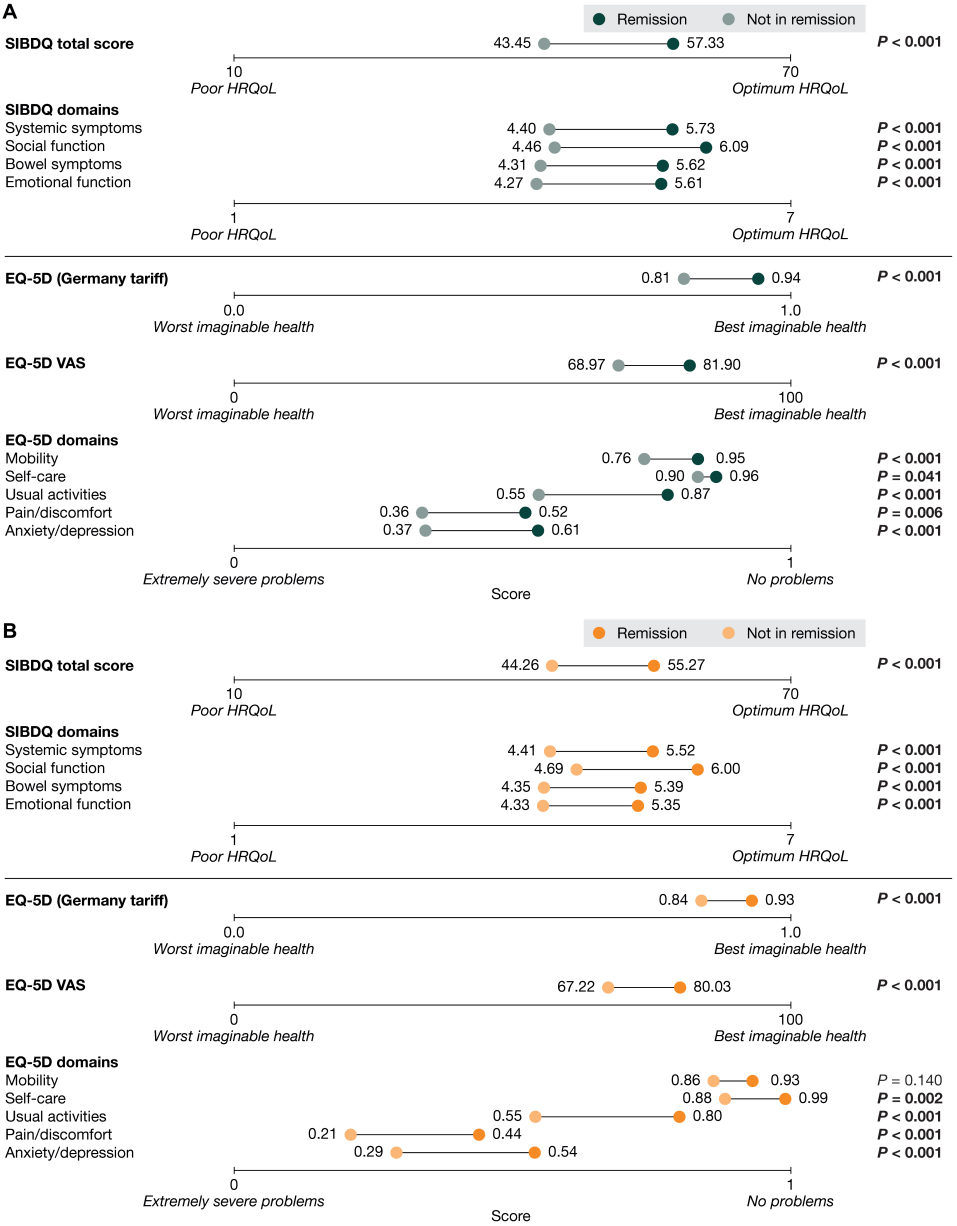
Short Inflammatory Bowel Disease Questionnaire (SIBDQ) and EQ-5D scores among patients in remission and not in remission for **(A)** patients with ulcerative colitis and **(B)** patients with Crohn’s disease. Data are shown for patients with complete data for all outcomes and potential confounding variables (for ulcerative colitis, n = 271 and n = 147 for those in remission and not in remission, respectively; for Crohn’s disease, n = 299 and n = 128 for those in remission and not in remission, respectively). The *P* values demonstrate the significance of differences in adjusted mean score between patients in remission and those not in remission; *P* values in bold indicate statistical significance (<. 05). Data were adjusted for age, sex, body mass index, time since inflammatory bowel disease diagnosis, time since the start of current treatment initiation, and disease severity at the time of current treatment initiation, using propensity score matching. HRQoL, health-related quality of life.

Adjusted mean SIBDQ, EQ-5D, and WPAI scores by remission status are shown in Figure 2. Among both patients with UC and patients with CD, those in remission generally demonstrated significantly better HRQoL than those not in remission. Among patients with UC, adjusted mean total SIBDQ scores were 57.33 and 43.45, and adjusted mean scores for SIBDQ domains ranged from 5.61 to 6.09 and from 4.27 to 4.46, for patients in remission and not in remission, respectively. Adjusted mean EQ-VAS scores were 81.90 and 58.97, and EQ-5D domain scores ranged from 0.52 to 0.96 and from 0.36 to 0.90, for patients in remission and not in remission, respectively. All measures were significantly different between those in remission and those not in remission (*P < *.05) (Figure 2A). Similarly, among patients with CD, adjusted mean total SIBDQ scores were 55.27 and 44.26, and adjusted mean scores for individual SIBDQ domains ranged from 5.35 to 6.00 and from 4.33 to 4.69, for patients in remission and not in remission, respectively. Adjusted mean scores for EQ-VAS were 80.03 and 67.22, and for individual EQ-5D domains ranged from 0.44 to 0.99 and from 0.21 to 0.88, for patients in remission and not in remission, respectively. All measures other than EQ-5D mobility were significantly different between those in remission and not in remission (*P* < .05) (Figure 2B). The sensitivity analysis among patients with CD in remission revealed that those with mucosal healing confirmed by endoscopy reported lower EQ-5D scores for the domain “usual activities” than patients with mucosal healing not confirmed by endoscopy (*P* = .044). There were no other differences in SIBDQ, EQ-5D, or WPAI scores among those in remission between those who had undergone endoscopy to confirm mucosal healing and those who had not undergone endoscopy (Supplementary Table 5).

**Figure 3.**
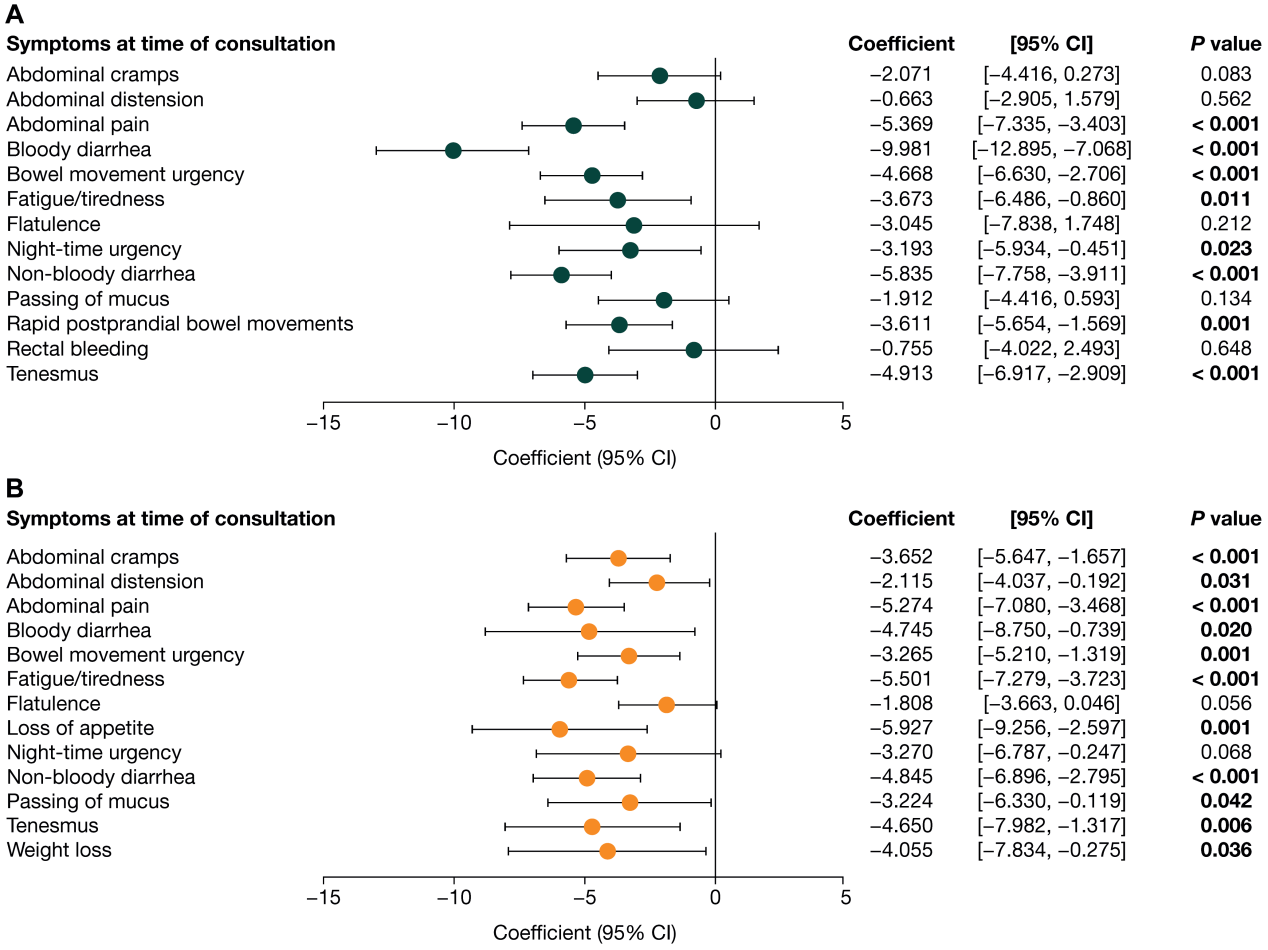
Association of symptoms with Short Inflammatory Bowel Disease Questionnaire (SIBDQ) scores in **(A)** patients with ulcerative colitis (n = 479) and **(B)** patients with Crohn’s disease (n = 509). Scores for the SIBDQ range from 10 to 70, with higher scores indicating better health-related quality of life. *P* values in bold indicate statistical significance (<. 05). CI, confidence interval.

**Figure 4.**
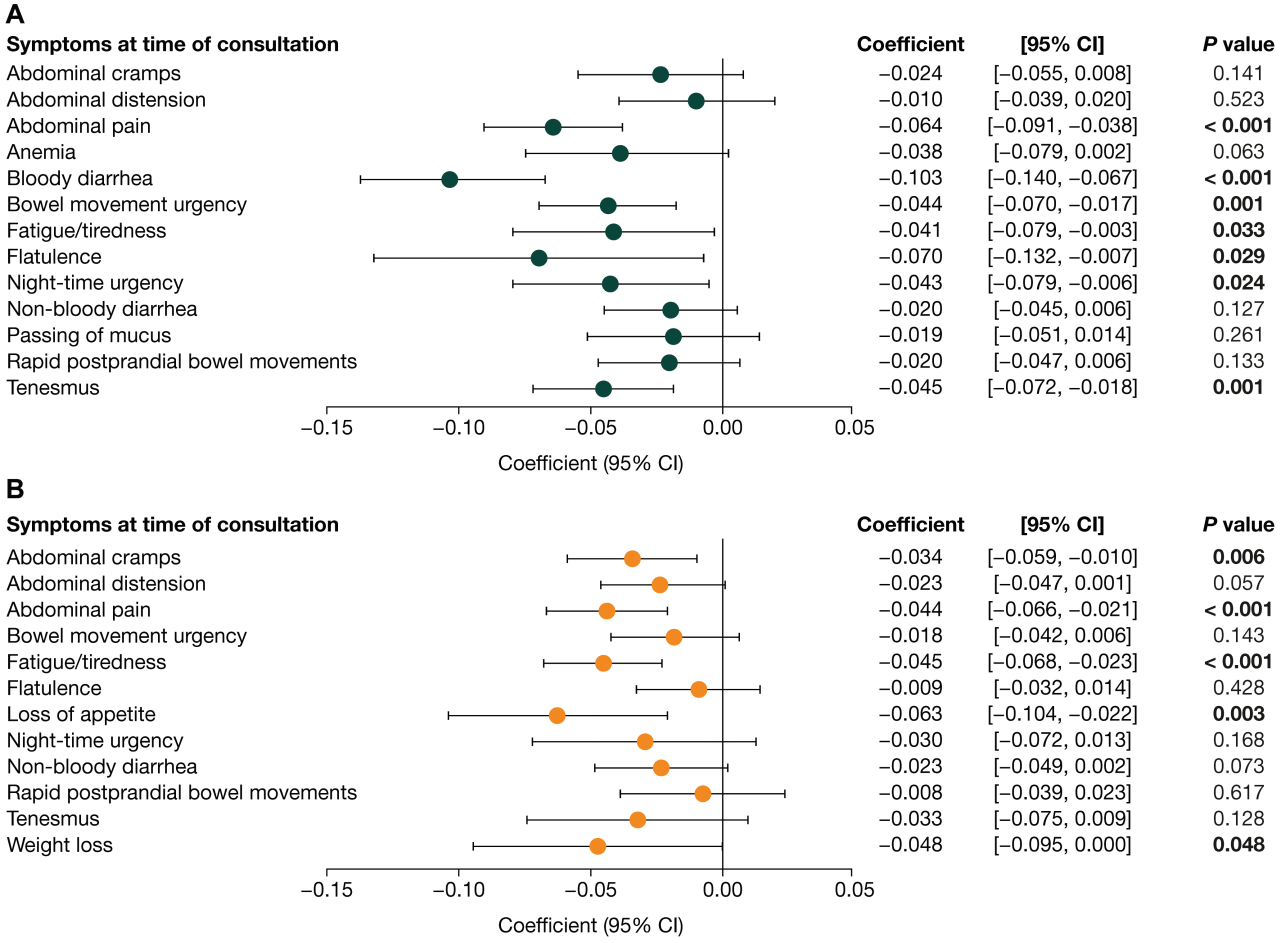
Association of symptoms with EQ-5D score in **(A)** patients with ulcerative colitis (n = 478) and **(B)** patients with Crohn’s disease (n = 509). Scores for the EQ-5D range from 0 to 1, with higher scores indicating better health-related quality of life (HRQoL). *P* values in bold indicate statistical significance (<. 05). CI, confidence interval.

**Figure 5.**
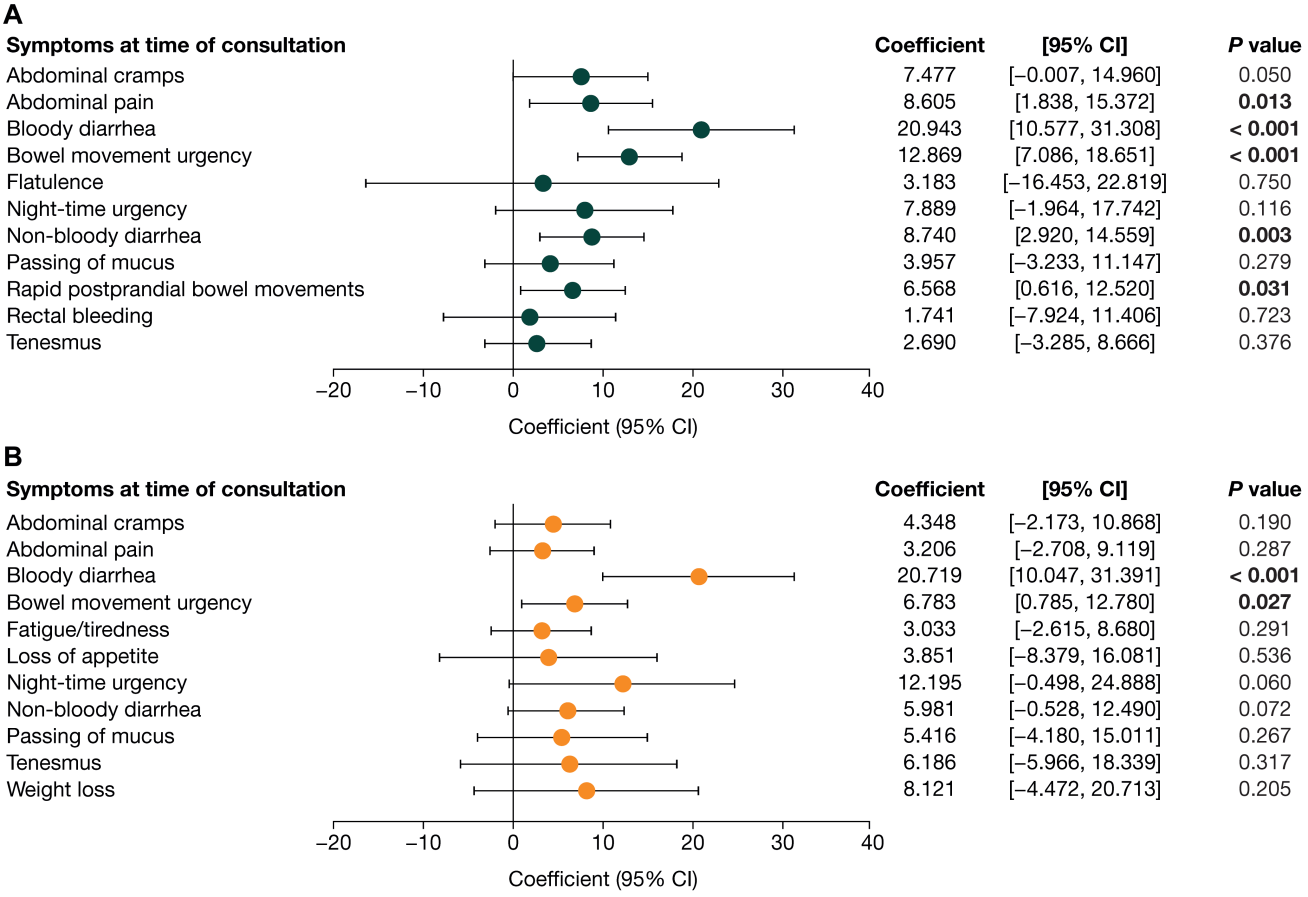
Association of symptoms with WPAI scores in **(A)** patients with ulcerative colitis (n = 235) and (**B)** patients with Crohn’s disease (n = 273). Data are given for patients currently working. Scores for the WPAI questionnaire range from 0% to 100%, with higher scores indicating greater impairment. *P* values in bold indicate statistical significance (<. 05). CI, confidence interval.

Impaired HRQoL was associated with high symptomatic burden. Among both patients with UC and patients with CD, lower (worse) SIBDQ scores were associated with patients experiencing abdominal pain, bloody and non-bloody diarrhea, bowel movement urgency, fatigue/tiredness, and tenesmus. Furthermore, night-time urgency and rapid postprandial bowel movements were associated with worse SIBDQ scores among patients with UC, and abdominal cramps, abdominal distension, loss of appetite, passing of mucus, and weight loss were all associated with worse SIBDQ scores among patients with CD (Figure 3). Lower (worse) EQ-5D scores were associated with abdominal pain and fatigue/tiredness among both patients with UC and patients with CD. In addition, worse EQ-5D scores were associated with bloody diarrhea, bowel movement urgency, flatulence, night-time urgency, and tenesmus in patients with UC, and with abdominal cramps, loss of appetite, and weight loss in patients with CD (Figure 4). Higher (worse) WPAI scores were associated with patients experiencing bloody diarrhea and bowel movement urgency, in both UC and CD. Moreover, abdominal pain, non-bloody diarrhea and rapid postprandial bowel movements were associated with worse WPAI scores among patients with UC (Figure 5).

### Treatment Satisfaction

Overall, 37.2% of patients with UC and 39.4% of patients with CD were not satisfied with their treatment at the time of consultation. In UC, 19.8% of patients in remission were not satisfied with their treatment, compared with 71.7% of those not in remission. Similarly, 27.9% of patients with CD in remission and 65.4% of those not in remission were not satisfied with their treatment (Supplementary Table 1).

**Figure 6.**
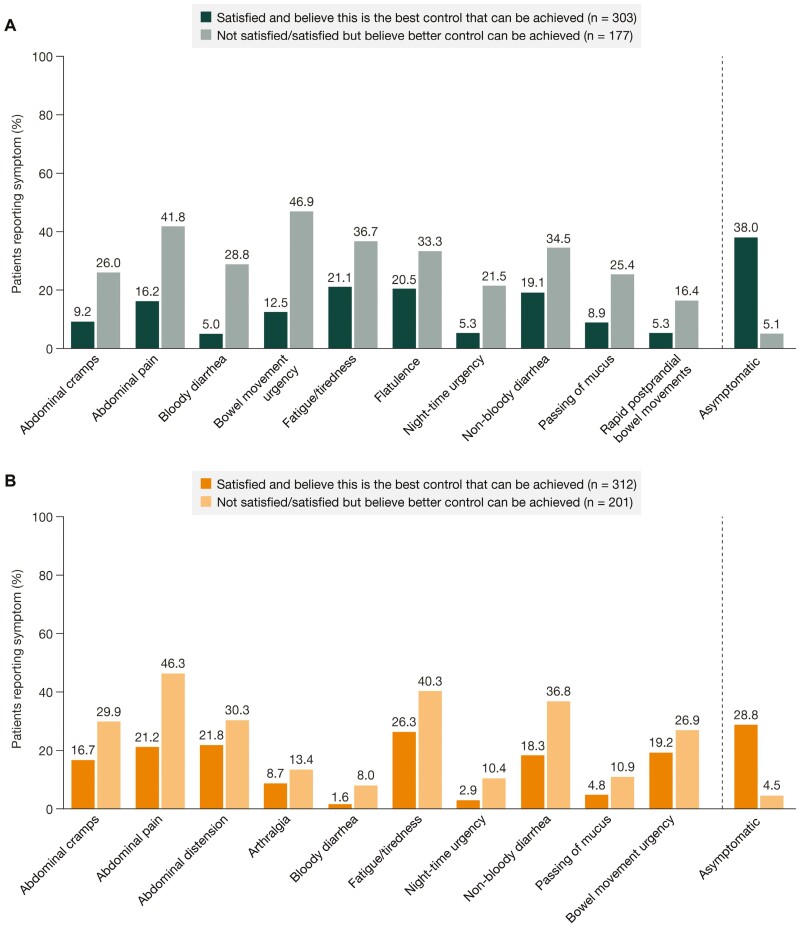
Proportions of patients with **(A)** ulcerative colitis and **(B)** Crohn’s disease, who experienced symptoms, among those who were satisfied with their treatment at the time of consultation and those who were not satisfied. Data are displayed for the 10 symptoms with the largest difference in the proportion of patients experiencing the symptom between those who were satisfied with their treatment at the time of consultation and those who were either not satisfied or were satisfied but believed that better control could be achieved. Data are also displayed for the proportion of patients who were asymptomatic.

Of those who were not satisfied with their treatment and had symptom data available, 94.9% (UC) and 95.5% (CD) were still reporting symptoms. The 10 symptoms with the largest difference in proportions of treatment-satisfied and treatment-dissatisfied patients reporting them are shown in Figure 6. The symptoms with the largest difference between the treatment-satisfied and treatment-dissatisfied groups were bowel movement urgency, abdominal pain, and bloody diarrhea (UC), and abdominal pain, non-bloody diarrhea, and fatigue/tiredness (CD).

## Discussion

This real-world, multinational study identified significant residual disease burden among European patients with moderate-to-severe IBD. We found that, although most patients were in remission, considerable proportions of patients with IBD were still reporting symptoms. These symptoms were significantly associated with reduced HRQoL. In line with this, a substantial proportion of patients were not satisfied with their treatment.

Among patients in remission, almost two-thirds of patients with UC and almost three-quarters of patients with CD were still experiencing symptoms, with abdominal pain, bowel movement urgency, and diarrhea being frequently reported. Previous studies have shown an increased prevalence of irritable bowel syndrome (IBS)-like symptoms, such as these, among patients with IBD in remission compared with the healthy population.^30,31^ Although it is possible that these symptoms relate to functional diseases such as IBS in some patients, they have been associated with residual endoscopic activity and increased risk of disease relapse.^30,31^ Indeed, our results show that a higher proportion of patients with UC who were later classified as not in remission reported experiencing IBS-like symptoms prior to initiation of their current treatment compared with those who were later classified as being in remission, including bowel movement urgency, flatulence, and rectal bleeding. Interestingly, no such trend was observed among patients with CD. Here, IBS-like symptoms were also associated with worse HRQoL and/or a greater impairment of normal activities. Another symptom that is worthy of note is fatigue/tiredness. This was reported by one of the largest proportions of patients across both UC and CD, both prior to initiation of current treatment and at the time of consultation, and among both those in remission and not in remission. Furthermore, it was identified as one of the most bothersome symptoms, and was significantly associated with decreased disease-specific and generic HRQoL. It is worth noting that, prior to initiation of current treatment, fatigue/tiredness was reported by a significantly higher proportion of patients who were later classified as being in remission, which may suggest differences in the burden of this symptom at baseline. Furthermore, while fatigue is a well-documented symptom of chronic diseases including IBD,^32,33^ it is not necessarily IBD specific, and its etiology is not well understood. Thus, it could have an unrelated underlying cause, such as mental health problems including anxiety or depression. However, the prevalence of fatigue in the population being studied (which was notably higher than that of depression and/or anxiety) and its significant correlation with multiple HRQoL outcomes suggest that it could be worth considering when developing treatment strategies.

Prior to initiation of current treatment, the majority of symptoms were reported by a similar proportion of patients who were later classified as being in remission or not in remission, across both UC and CD. At the time of consultation, patients with UC in remission and not in remission were both still reporting residual symptoms. Remission was generally associated with a significantly reduced probability of experiencing most symptoms compared with those not in remission, including bloody diarrhea, bowel movement urgency, and passing of mucus. However, some symptoms, including flatulence and abdominal distension, were not significantly less likely to occur in patients in remission compared with those not in remission. These symptoms reflect limitations of current treatments, and were associated with impaired generic and disease-specific HRQoL in this study. In patients with CD, there were even fewer differences in the proportion of patients reporting symptoms at the time of consultation between those in remission and not in remission compared with UC. Only 4 symptoms were significantly less likely to be reported by patients in remission than those not in remission: abdominal pain, non-bloody diarrhea, fatigue/tiredness, and loss of appetite. Many symptoms, including those identified as bothersome and/or closely associated with impaired HRQoL, are not significantly impacted by achievement of remission. These symptoms include bloody diarrhea, flatulence, and abdominal cramps. This may suggest insufficient disease control and residual disease activity among patients in remission, particularly in CD. These data may therefore suggest that a more comprehensive approach for the classification of disease activity should be employed, which better reflects patient experiences and considers additional factors such as extraintestinal manifestations and HRQoL.

Occurrence of symptoms was significantly associated with worse disease-specific and generic HRQoL. It was therefore not surprising that, while patients with UC and CD in remission generally had significantly better HRQoL than those not in remission, their SIBDQ and EQ-5D scores still suggested impaired HRQoL among both patients in remission and not in remission compared with the general population. The estimated cutoff for an SIBDQ score indicative of disease-free HRQoL is 56.^34^ In this study, the adjusted mean scores were 43.45 (UC, not in remission), 57.33 (UC, in remission), 44.26 (CD, not in remission), and 55.27 (CD, in remission). As the mean scores are very close to this threshold, a considerable number of patients, even those in remission, are likely to be falling short of this definition. This indicates limitations of current treatments in terms of their ability to fully restore HRQoL. This may be due, at least in part, to residual symptoms such as diarrhea, bowel movement urgency, and fatigue/tiredness. Similarly, the adjusted mean scores for the EQ-VAS in different subgroups of patients with IBD in this study (67.22-81.90) were lower than that of the healthy, age-matched European population norm (~85-90).^35-37^ Patients in this study were also more likely to select responses in the EQ-5D dimensions indicative of moderate or severe problems with normal functioning than the healthy population.^36,37^ Overall, these data indicate that both patients in remission and not in remission experience impaired HRQoL.

The impaired HRQoL experienced by patients in remission suggests that HRQoL is being influenced by components of disease that are not accounted for in the current definition of remission. As suggested by their association with worse EQ-5D and SIBDQ scores, this may include nongastrointestinal symptoms such as fatigue/tiredness and loss of appetite. Thus, it is possible that the accepted definitions of clinical remission (including normalization of bowel habits and endoscopically confirmed mucosal healing) are of limited benefit to many patients. Patients and clinicians may benefit from broader assessment of disease activity, for example, by routinely assessing disease-specific and generic HRQoL. Better still, more comprehensive efficacy measures that integrate multiple variables would allow for holistic assessment of disease activity using a single endpoint. To this end, a novel endpoint has recently been proposed, termed comprehensive disease control.^38^ This encompasses improvements in symptoms, inflammatory burden, endoscopic activity, and HRQoL. Such complex, multidimensional measures could better reflect the current disease burden, and will likely provide more detailed and useful information on the efficacy profiles of IBD treatments. Indeed, a consensus process identified symptoms (including extraintestinal manifestations), HRQoL, and objective measures of disease activity such as endoscopic activity and inflammatory biomarker concentration to be important aspects of comprehensive disease control.^39^ Full validation and evaluation of the anatomy of this endpoint is underway and will be published in due course.

Our data may also indicate more general differences in disease pathology between UC and CD. In patients with UC, those in remission had significantly better HRQoL and likelihood of symptoms than those not in remission. In contrast, although patients with CD in remission reported significantly better HRQoL vs those not in remission, they did not generally experience an improvement in likelihood of symptoms. This indicates complex and multifactorial disease burden and suggests that, particularly in CD, there are factors contributing to overall HRQoL other than the likelihood of experiencing a symptom. These may include symptom severity and unpredictability, ability to manage symptoms, mental health, disease duration, and external responses to disease such as societal understanding and flexibility of employers.^40,41^

It should be highlighted that this study included patients from France, Germany, Italy, and Spain who likely have access to more advanced therapies than patients in other European countries and other parts of the world. Indeed, more than half of patients with IBD were receiving advanced treatment. Therefore, it is likely that these data underestimate the scale of residual disease burden among patients with IBD worldwide. Furthermore, of patients who were not in remission, a significant proportion were receiving advanced treatment (UC 68.8%; CD 54.9%). This may demonstrate considerable therapeutic gaps in the management of patients with IBD, rather than physician hesitance to administer advanced therapies.

The strengths of this study include the real-world, DSP methodology, which is highly robust, providing information from both physician and patient perspectives.^18,20-24^ The multinational setting, the generation of the study population from consecutive consulting patients, and use of PSM reduce bias and increase the validity of the conclusions. A limitation is that the patient and physician populations are not truly random because the patient population would have been influenced by factors including the frequency of primary care visits, and the physician population would have been influenced by their willingness to take part. In addition, the DSP inclusion criteria for UC was more stringent than for CD, and physician categorization of patients’ IBD as moderate to severe was done per local clinical guidelines, which may include subjective components. Furthermore, no adjustments for multiple testing were made, and so caution should be used when interpreting the results. It is also worth acknowledging the heterogeneity in the patient population, owing to the real-world, observational study design. For example, patients with underlying mental health problems, including anxiety and depression, were not excluded from this study. While this could skew the results due to the established link between mental health problems and IBD,^42,43^ the inclusion of these patients may increase the generalizability of findings. In addition, the majority of patients were not affected by anxiety or depression. Furthermore, PSM allowed for balancing of confounding factors that may have otherwise skewed the results. To further increase the validity of the results, it may have been beneficial to perform PSM on more variables, for example, by collecting data on non-IBD medications such as antidepressants. In addition, future real-world evaluation of unmet needs in IBD would benefit from formal longitudinal assessment of the effect of specific medications on disease burden.

## Conclusions

Although achieving remission is important in IBD, the goal from the patient’s perspective is elimination of symptoms and restoration of optimum HRQoL. This study showed that the majority of patients with IBD, including those who are in remission, are still reporting symptoms, and HRQoL is impaired relative to the general population. In line with this, a high proportion of patients are not satisfied with their treatment. This highlights the need for therapies that provide more comprehensive disease control,^38^ and novel efficacy endpoints that encompass aspects like HRQoL and symptoms to better assess them.

## Supplementary Material

izae119_suppl_Supplementary_Material
