# Supplementary material for: Residual Disease Burden Among European Patients With Inflammatory Bowel Disease: A Real-World Survey
Source: Inflamm Bowel Dis. 2024 Jun 7;31(2):411–24. doi: 10.1093/ibd/izae119 (PMC11808571; doi:10.1093/ibd/izae119)
Supplement: izae119_suppl_Supplementary_Material [file izae119_suppl_supplementary_material.docx]

**Residual Disease Burden Among European Patients With Inflammatory Bowel Disease: A Real World Survey**

Johan Burisch, Ailsa Hart, Andreas Sturm, Christine Rudolph, Rachael Meadows, Anna Jus, Fatima Dawod, Haridarshan Patel, and Alessandro Armuzzi

Supplementary Material

**Supplementary Table 1.** Patient demographics and clinical characteristics.

| **Variable** | **Patients with UC**  **n = 502** | **Patients with CD**  **n = 538** |
| --- | --- | --- |
| *Demographics* | | |
| **Age, years** |  |  |
| Mean ± SD | 38.9 ± 13.5 | 38.3 ± 12.6 |
| **Sex** |  |  |
| Male, n (%) | 231 (46.0) | 288 (53.5) |
| Female, n (%) | 271 (54.0) | 250 (46.5) |
| **Ethnicity** |  |  |
| White, n (%) | 485 (96.6) | 516 (95.9) |
| Other, n (%) | 17 (3.4) | 22 (4.1) |
| **BMI, kg/m^2^** |  |  |
| Mean ± SD | 23.3 ± 3.4 | 23.5 ± 3.5 |
| **Employment status** | **n = 491** | **n = 533** |
| In full-time employment, n (%) | 289 (58.9) | 298 (55.9) |
| In part-time employment, n (%) | 47 (9.6) | 54 (10.1) |
| On long-term sick leave, n (%) | 12 (2.4) | 9 (1.7) |
| Homemaker, n (%) | 38 (7.7) | 35 (6.6) |
| Student, n (%) | 55 (11.2) | 55 (10.3) |
| Retired, n (%) | 38 (7.7) | 33 (6.2) |
| Unemployed, n (%) | 12 (2.4) | 49 (9.2) |
| **Number of days taken off work in last 3 months owing to IBD** | **n = 104** | **n = 114** |
| Mean ± SD | 8.5 ± 9.7 | 7.7 ± 14.4 |
| **Impact of IBD on employment** |  |  |
| Worked fewer hours, n (%) | 55 (11.0) | 58 (10.8) |
| Stopped working/retired, n (%) | 49 (9.8) | 60 (11.2) |
| *Clinical characteristics* |  |  |
| **Disease duration, years** | **n = 456** | **n = 489** |
| Mean ± SD | 5.2 ± 5.8 | 6.0 ± 7.1 |
| **Time from symptom onset to first consultation, months** | **n = 476** | **n = 502** |
| Mean ± SD | 4.1 ± 9.4 | 6.0 ± 16.6 |
| **Time from first consultation to diagnosis, months** | **n = 415** | **n = 443** |
| Mean ± SD | 2.2 ± 5.4 | 5.2 ± 19.3 |
| **Previous misdiagnoses** | **n = 497** | **n = 536** |
| Yes, n (%) | 102 (20.5) | 149 (27.8) |
| No, n (%) | 395 (79.5) | 387 (72.2) |
| **Disease severity at initiation of treatment** |  |  |
| Moderate, n (%) | 343 (68.3) | 384 (71.4) |
| Severe, n (%) | 159 (31.7) | 154 (28.6) |
| **Disease severity at time of consultation** |  |  |
| Mild, n (%) | 342 (68.1) | 370 (68.8) |
| Moderate, n (%) | 137 (27.3) | 157 (29.2) |
| Severe, n (%) | 23 (4.6) | 11 (2.0) |
| **Current symptoms** | **n = 487** | **n = 519** |
| Symptomatic, n (%) | 363 (74.5) | 420 (80.9) |
| Asymptomatic, n (%) | 124 (25.5) | 99 (19.1) |
| **Lines of treatment received** | **n = 399** | **n = 538** |
| 0, n (%) | 0 | 1 (0.2) |
| 1, n (%) | 93 (23.3) | 156 (29.0) |
| 2, n (%) | 175 (43.9) | 215 (40.0) |
| 3, n (%) | 70 (17.5) | 113 (21.0) |
| 4 or more, n (%) | 61 (15.3) | 53 (9.9) |
| **In remission, n (%)** | 332 (66.1) | 374 (69.5) |
| **Symptoms** | **n = 322** | **n = 359** |
| Symptomatic, n (%) | 205 (63.7) | 266 (74.1) |
| Asymptomatic, n (%) | 117 (36.3) | 93 (25.9) |
| **Treatment satisfaction^a^** | **n = 328** | **n = 369** |
| Satisfied, n (%) | 263 (80.2) | 266 (72.1) |
| Not satisfied, n (%) | 65 (19.8) | 103 (27.9) |
| **Concomitant conditions** |  |  |
| Anxiety, n (%) | 27 (8.1) | 32 (8.6) |
| Depression, n (%) | 11 (3.3) | 15 (4.0) |
| **Not in remission, n (%)** | 170 (33.9) | 164 (30.5) |
| **Symptoms** | **n = 165** | **n = 160** |
| Symptomatic, n (%) | 158 (95.8) | 154 (96.2) |
| Asymptomatic, n (%) | 7 (4.2) | 6 (3.8) |
| **Treatment satisfaction^b^** | **n = 166** | **n = 162** |
| Satisfied, n (%) | 47 (28.3) | 56 (34.6) |
| Not satisfied, n (%) | 119 (71.7) | 106 (65.4) |
| **Concomitant conditions** |  |  |
| Anxiety, n (%) | 29 (17.1) | 34 (20.7) |
| Depression, n (%) | 15 (8.8) | 27 (16.5) |
| **Treatment at time of consultation** |  |  |
| 5-ASA, n (%) | 254 (50.6) | 183 (34.0) |
| Corticosteroid, n (%) | 86 (17.1) | 117 (21.7) |
| Immunomodulator, n (%) | 107 (21.3) | 132 (24.5) |
| Biologic or biosimilar anti-TNF, n (%) | 231 (46.0) | 233 (43.3) |
| Biologic anti-integrin, n (%) | 49 (9.8) | 39 (7.2) |
| Anti-IL-12/23 inhibitor, n (%) | 13 (2.6) | 35 (6.5) |
| JAK inhibitor, n (%) | 11 (2.2) | 0 |

For a given variable, cohort size is indicated if data were not available for all patients.

BMI, body mass index; CD, Crohn’s disease; IBD, Inflammatory bowel disease; IL, interleukin; JAK, Janus kinase; SD, standard deviation; TNF, tumor necrosis factor; UC, ulcerative colitis; 5-ASA, 5-aminosalicylates.

^a^Satisfied patients are those who described themselves as satisfied with their treatment at the time of consultation and believed that this was the best control that could be achieved. Not satisfied patients are those who described themselves as either not satisfied with their treatment at the time of consultation or were satisfied but believed better control could be achieved.

**Supplementary Table 2.** Results of PSM on baseline covariates for patients with UC and CD.

|  | **Before matching** | | | **PSM** | | |
| --- | --- | --- | --- | --- | --- | --- |
| **Covariate** | **In remission** | **Not in remission** | **SMD (%)** | **In remission** | **Not in remission** | **SMD (%)** |
| **Patients with UC** | **n = 271** | **n = 147** |  | **n = 271** | **n = 147** |  |
| Age, years, mean | 37.6 | 38.1 | 3.6 | 37.9 | 38.2 | 1.7 |
| Sex, female, % | 51.7 | 61.9 | 20.7 | 57.4 | 56.2 | −2.4 |
| BMI, kg/m^2^, mean | 23.3 | 23.0 | −9.5 | 23.4 | 23.6 | 6.0 |
| Disease duration, years, mean | 5.0 | 5.2 | 3.4 | 5.1 | 5.3 | 3.4 |
| Severity before current treatment, severe, % | 31.7 | 36.1 | 9.1 | 33.7 | 31.6 | −4.6 |
| Duration of current treatment regimen, days, mean | 741 | 392 | −44.1 | 674 | 646 | −3.0 |
| **Patients with CD** | **n = 128** | **n = 299** |  | **n = 128** | **n = 299** |  |
| Age, years, mean | 37.4 | 37.3 | −0.2 | 38.7 | 38.4 | −2.7 |
| Sex, female, % | 45.3 | 45.8 | 1.0 | 47.1 | 45.9 | −2.4 |
| BMI, kg/m^2^, mean | 23.3 | 23.3 | 1.1 | 23.3 | 23.4 | 3.9 |
| Disease duration, years, mean | 5.2 | 6.0 | 12.4 | 6.2 | 6.2 | 0.5 |
| Severity prior to current treatment, severe, % | 28.9 | 29.4 | 1.2 | 32.8 | 28.6 | -9.1 |
| Duration of current treatment regimen, days, mean | 14.2 | 24.6 | 40.9 | 19.8 | 21.8 | 8.0 |

BMI, body mass index; CD, Crohn’s disease; PSM, propensity score matching; SMD, standardized mean difference; UC, ulcerative colitis.

**Supplementary Table 3.** Demographics and clinical characteristics for patients with CD in remission, among those who had undergone endoscopy to confirm mucosal healing and those who had not undergone endoscopy.

|  | **Remission with mucosal healing not confirmed by endoscopy**  **n = 58** | **Remission with mucosal healing confirmed by endoscopy**  **n = 303** | ***P* value** |
| --- | --- | --- | --- |
| **Patient demographics** |  |  |  |
| Age, years |  |  |  |
| Mean, years ± SD | 40.1 ± 12.5 | 38.6 ± 12.6 | 0.399 |
| Sex |  |  |  |
| Male, n (%) | 30 (51.7) | 150 (49.5) | 0.776 |
| Ethnicity |  |  |  |
| White, n (%) | 57 (98.3) | 290 (95.7) | 0.257 |
| BMI |  |  |  |
| Mean, kg/m^2^ ± SD | 24.5 ± 3.8 | 23.7 ± 3.5 | 0.118 |
| **Clinical characteristics** |  |  |  |
| Disease duration | n = 48 | n = 287 |  |
| Mean, years ± SD | 5.7 ± 4.5 | 6.1 ± 5.9 | 0.699 |
| Time from onset of symptoms to first consultation | n = 22 | n = 87 |  |
| Mean, months ± SD | 4.5 ± 4.6 | 5.3 ± 14.0 | 0.789 |
| Time from first consultation to diagnosis | n = 46 | n = 276 |  |
| Mean, weeks ± SD | 25.1 ± 50.2 | 14.9 ± 41.7 | 0.136 |
| Disease severity at initiation of treatment |  |  |  |
| Moderate, n (%) | 43 (74.1) | 197 (65.0) | 0.178 |
| Severe, n (%) | 15 (25.9) | 106 (35.0) |  |
| Disease severity at time of consultation |  |  |  |
| Mild, n (%) | 49 (84.5) | 267 (88.1) | 0.450 |
| Moderate, n (%) | 9 (15.5) | 35 (11.6) |  |
| Severe, n (%) | 0 (0.0) | 1 (0.3) |  |

*P* values in bold indicate statistical significance (< .05).

Data are shown for participants who were classed as being in remission and had full symptom data available (n = 361).

BMI, body mass index; CD, Crohn’s disease; SD, standard deviation.

**Supplementary Table 4.** Symptoms and their bothersomeness.

| **Symptoms at time of consultation, n (%)** | **Patients with UC** | | **Patients with CD** | |
| --- | --- | --- | --- | --- |
|  | **Symptom occurrence** | **Bothersomeness** | **Symptom occurrence** | **Bothersomeness** |
|  | **n = 487** | **n = 403** | **n = 519** | **n = 450** |
| Abdominal cramps | 76 (15.6) | 81 (20.1) | 112 (21.6) | 109 (24.2) |
| Abdominal distension | 83 (17.0) | 56 (13.9) | 130 (25.0) | 109 (24.2) |
| Abdominal pain | 125 (25.7) | 93 (23.1) | 163 (31.4) | 143 (31.8) |
| Anemia | 18 (3.7) | 7 (1.7) | 18 (3.5) | 9 (2.0) |
| Arthralgia | 26 (5.3) | 19 (4.7) | 54 (10.4) | 33 (7.3) |
| Back pain | 33 (6.8) | 12 (3.0) | 23 (4.4) | 11 (2.4) |
| Bloody diarrhea | 67 (13.8) | 113 (28.0) | 21 (4.0) | 62 (13.8) |
| Bowel movement urgency | 124 (25.5) | 123 (30.5) | 115 (22.2) | 101 (22.4) |
| Constipation | 9 (1.8) | 7 (1.7) | 17 (3.3) | 13 (2.9) |
| Fatigue/tiredness | 131 (26.9) | 101 (25.1) | 163 (31.4) | 144 (32.0) |
| Fever | 4 (0.8) | 0 (0.0) | 6 (1.2) | 1 (0.2) |
| Flatulence | 123 (25.3) | 92 (22.8) | 141 (27.2) | 109 (24.2) |
| Joint swelling | 7 (1.4) | 3 (0.7) | 10 (1.9) | 4 (0.9) |
| Loss of appetite | 35 (7.2) | 19 (4.7) | 38 (7.3) | 11 (2.4) |
| Night sweats | 16 (3.3) | 7 (1.7) | 14 (2.7) | 2 (0.4) |
| Night-time urgency | 56 (11.5) | 61 (15.1) | 31 (6.0) | 39 (8.7) |
| Non-bloody diarrhea | 123 (25.3) | 86 (21.3) | 135 (26.0) | 135 (30.0) |
| Passing of mucus | 75 (15.4) | 42 (10.4) | 37 (7.1) | 19 (4.2) |
| Rapid postprandial bowel movements | 47 (9.7) | 28 (6.9) | 66 (12.7) | 51 (11.3) |
| Rectal bleeding | 40 (8.2) | 48 (11.9) | 12 (2.3) | 31 (6.9) |
| Tenesmus | 46 (9.4) | 41 (10.2) | 32 (6.2) | 29 (6.4) |
| Vomiting/nausea | 14 (2.9) | 6 (1.5) | 20 (3.9) | 13 (2.9) |
| Weight loss | 27 (5.5) | 11 (2.7) | 28 (5.4) | 19 (4.2) |
| Other | 6 (1.2) | 7 (1.7) | 6 (1.2) | 5 (1.1) |

Symptom occurrence describes the number and proportion of patients reporting a given symptom at the time of consultation. Bothersomeness describes the number of patients who listed a given symptom when they were asked to select their 3 most bothersome symptoms at the time of consultation.

Abbreviations: CD, Crohn’s disease; UC, ulcerative colitis.

**Supplementary Table 5.** Symptoms and PROs for patients with CD in remission, among those who had undergone endoscopy to confirm mucosal healing and those who had not undergone endoscopy.

|  | **Remission with mucosal healing not confirmed by endoscopy**  **n = 58** | **Remission with mucosal healing confirmed by endoscopy**  **n = 303** | ***P* value** |
| --- | --- | --- | --- |
| **Symptoms** |  |  |  |
| Number of patients with symptoms | n = 58 | n = 302 |  |
| Abdominal cramps, n (%) | 4 (6.9) | 26 (8.6) | 0.800 |
| Abdominal distension (bloating), n (%) | 9 (15.5) | 44 (14.6) | 0.841 |
| Abdominal pain, n (%) | 5 (8.6) | 69 (22.8) | **0.013** |
| Anal discharge/passing of mucus, n (%) | 2 (3.4) | 4 (1.3) | 0.249 |
| Anemia, n (%) | 3 (5.2) | 25 (8.3) | 0.594 |
| Anorexia, n (%) | 1 (1.7) | 3 (1.0) | 0.506 |
| Arthralgia, n (%) | 1 (1.7) | 19 (6.3) | 0.220 |
| Back pain, n (%) | 0 (0.0) | 6 (2.0) | 0.595 |
| Bowel movement urgency, n (%) | 2 (3.4) | 19 (6.3) | 0.549 |
| Colic, n (%) | 1 (1.7) | 9 (3.0) | 1.000 |
| Constipation, n (%) | 1 (1.7) | 12 (4.0) | 0.702 |
| Diarrhea – bloody, n (%) | 1 (1.7) | 6 (2.0) | 1.000 |
| Diarrhea – non-bloody, n (%) | 6 (10.3) | 22 (7.3) | 0.424 |
| Fatigue, n (%) | 5 (8.6) | 61 (20.2) | **0.041** |
| Flatulence, n (%) | 6 (10.3) | 38 (12.6) | 0.827 |
| Joint swelling, n (%) | 0 (0.0) | 2 (0.7) | 1.000 |
| Loss of appetite, n (%) | 1 (1.7) | 9 (3.0) | 1.000 |
| Night sweats, n (%) | 0 (0.0) | 2 (0.7) | 1.000 |
| Night-time bowel movement urgency, n (%) | 1 (1.7) | 1 (0.3) | 0.297 |
| Osteopenia, n (%) | 0 (0.0) | 1 (0.3) | 1.000 |
| Rapid postprandial bowel movements, n (%) | 1 (1.7) | 11 (3.6) | 0.699 |
| Rectal bleeding, n (%) | 1 (1.7) | 3 (1.0) | 0.506 |
| Tenesmus, n (%) | 1 (1.7) | 3 (1.0) | 0.506 |
| Vomiting/nausea, n (%) | 0 (0.0) | 1 (0.3) | 1.000 |
| Weight gain, n (%) | 2 (3.4) | 3 (1.0) | 0.185 |
| Weight loss, n (%) | 1 (1.7) | 8 (2.6) | 1.000 |
| **PROs** |  |  |  |
| SIBDQ - Systemic symptoms score | n = 25 | n = 89 |  |
| Mean ± SD | 5.74 ± 1.49 | 5.68 ± 1.37 | 0.849 |
| SIBDQ - Social function score | n = 24 | n = 89 |  |
| Mean ± SD | 6.08 ± 1.57 | 6.11 ± 1.11 | 0.934 |
| SIBDQ - Bowel symptoms score | n = 25 | n = 89 |  |
| Mean ± SD | 5.76 ± 1.39 | 5.71 ± 1.03 | 0.848 |
| SIBDQ - Emotional function score | n = 24 | n = 89 |  |
| Mean ± SD | 5.75 ± 1.28 | 5.60 ± 1.18 | 0.595 |
| Total SIBDQ score | n = 24 | n = 89 |  |
| Mean ± SD | 58.5 ± 13.24 | 57.52 ± 10.04 | 0.693 |
| EQ-5D - Mobility | n = 25 | n = 88 |  |
| Mean ± SD | 1.16 ± 0.55 | 1.16 ± 0.48 | 0.994 |
| EQ-5D - Self-care | n = 24 | n = 88 |  |
| Mean ± SD | 1.08 ± 0.41 | 1.03 ± 0.18 | 0.389 |
| EQ-5D - Usual activities | n = 25 | n = 88 |  |
| Mean ± SD | 1.36 ± 0.76 | 1.14 ± 0.38 | **0.044** |
| EQ-5D - Pain/discomfort | n = 25 | n = 88 |  |
| Mean ± SD | 1.60 ± 0.82 | 1.53 ± 0.64 | 0.671 |
| EQ-5D - Anxiety/depression | n = 25 | n = 88 |  |
| Mean ± SD | 1.56 ± 0.92 | 1.50 ± 0.68 | 0.720 |
| EQ-5D 5L utility score (Italy tariff) | n = 24 | n = 88 |  |
| Mean ± SD | 0.92 ± 0.12 | 0.93 ± 0.07 | 0.604 |
| EQ-5D 5L utility score (UK tariff) | n = 24 | n = 88 |  |
| Mean ± SD | 0.85 ± 0.23 | 0.87 ± 0.13 | 0.634 |
| EQ-5D 5L utility score (France tariff) | n = 24 | n = 88 |  |
| Mean ± SD | 0.94 ± 0.16 | 0.96 ± 0.07 | 0.298 |
| EQ-5D 5L utility score (Germany tariff) | n = 24 | n = 88 |  |
| Mean ± SD | 0.91 ± 0.20 | 0.94 ± 0.09 | 0.302 |
| EQ-5D 5L utility score (Spain tariff) | n = 24 | n = 88 |  |
| Mean ± SD | 0.89 ± 0.19 | 0.90 ± 0.11 | 0.598 |
| EQ-VAS | n = 25 | n = 88 |  |
| Mean ± SD | 82.04 ± 13.58 | 81.09 ± 12.09 | 0.737 |
| WPAI - Absenteeism | n = 17 | n = 59 |  |
| Mean ± SD | 4.24 ± 11.14 | 1.09 ± 3.09 | 0.054 |
| WPAI - Presenteeism | n = 17 | n = 61 |  |
| Mean ± SD | 13.53 ± 15.79 | 11.97 ± 17.11 | 0.736 |
| WPAI - Overall work impairment | n = 17 | n = 58 |  |
| Mean ± SD | 16.16 ± 19.86 | 13.35 ± 17.61 | 0.576 |
| WPAI - Overall activity impairment | n = 25 | n = 87 |  |
| Mean ± SD | 13.20 ± 14.92 | 14.83 ± 19.70 | 0.703 |

*P* values in bold indicate statistical significance (< .05).

Data are shown for participants who were classed as being in remission and had full symptom data available (n = 361).

CD, Crohn’s disease; PRO, patient-reported outcome; SD, standard deviation; SIBDQ, Short Inflammatory Bowel Disease Questionnaire; WPAI, work productivity and activity impairment

**Supplementary Figure 1.** Proportion of patients who reported experiencing each symptom before initiation of their current treatment among patients in remission and not in remission at the time of their consultation in **a)** patients with UC and **b)** patients with CD.


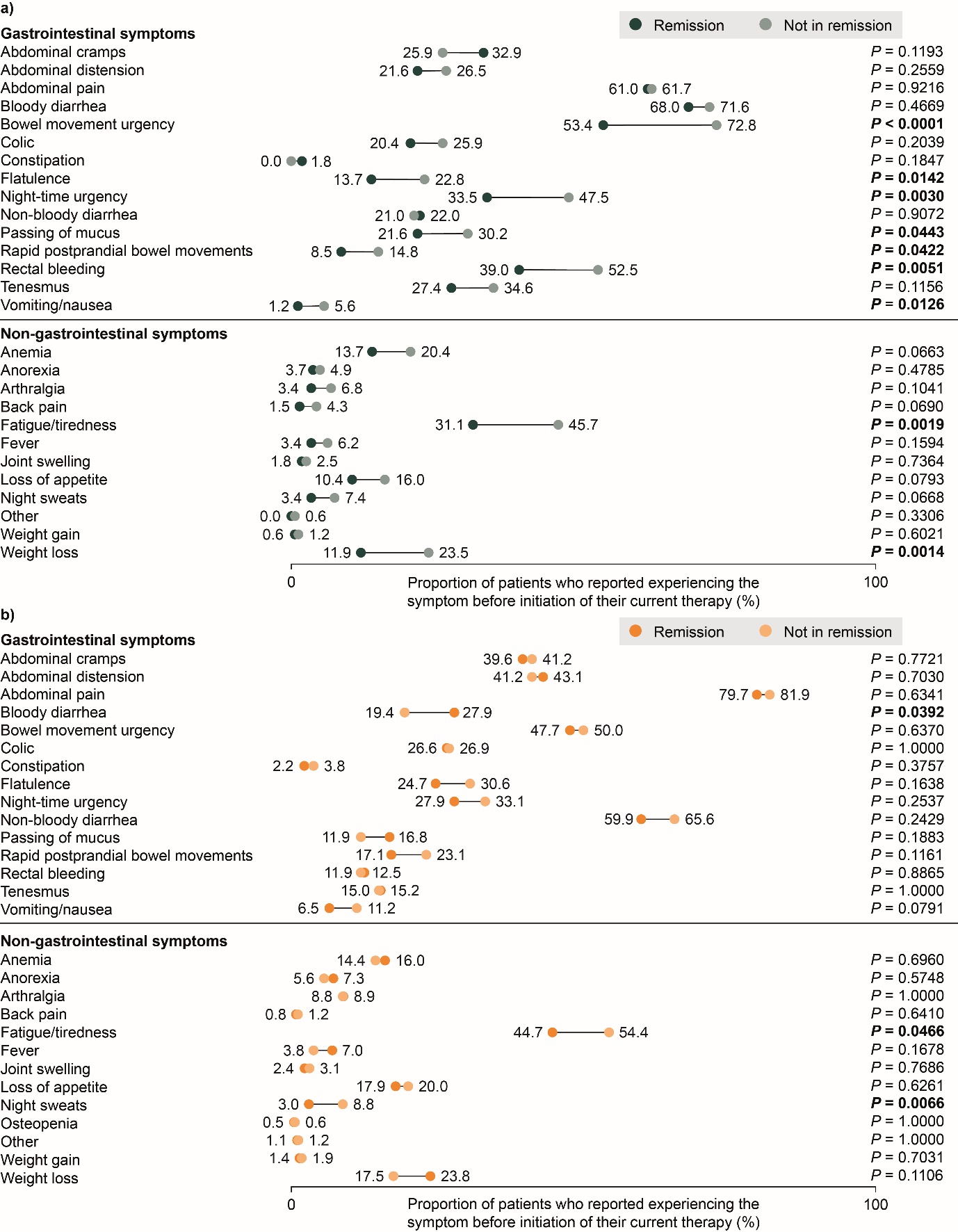


Data are shown for patients with complete data for all outcomes (for UC, n = 332 and n = 170 for those in remission and not in remission, respectively; for CD, n = 374 and n = 164 for those in remission and not in remission, respectively). The *P* values demonstrate the significance of differences in the proportion of patients who reported each symptom before initiation of their current therapy between patients in remission and those not in remission; *P* values in bold indicate statistical significance (< .05).

CD, Crohn’s disease; UC, ulcerative colitis.
